# Supplementary material for: Human Endogenous Retrovirus (HERV)-K env Gene Knockout Affects Tumorigenic Characteristics of nupr1 Gene in DLD-1 Colorectal Cancer Cells
Source: Int J Mol Sci. 2021 Apr 11;22(8):3941. doi: 10.3390/ijms22083941 (PMC8070087; doi:10.3390/ijms22083941)
Supplement: Supplementary file 1 [file ijms-22-03941-s001.pdf]

HERV-K119 chr12:58,327,625-58,336,907 (-) (hg38)

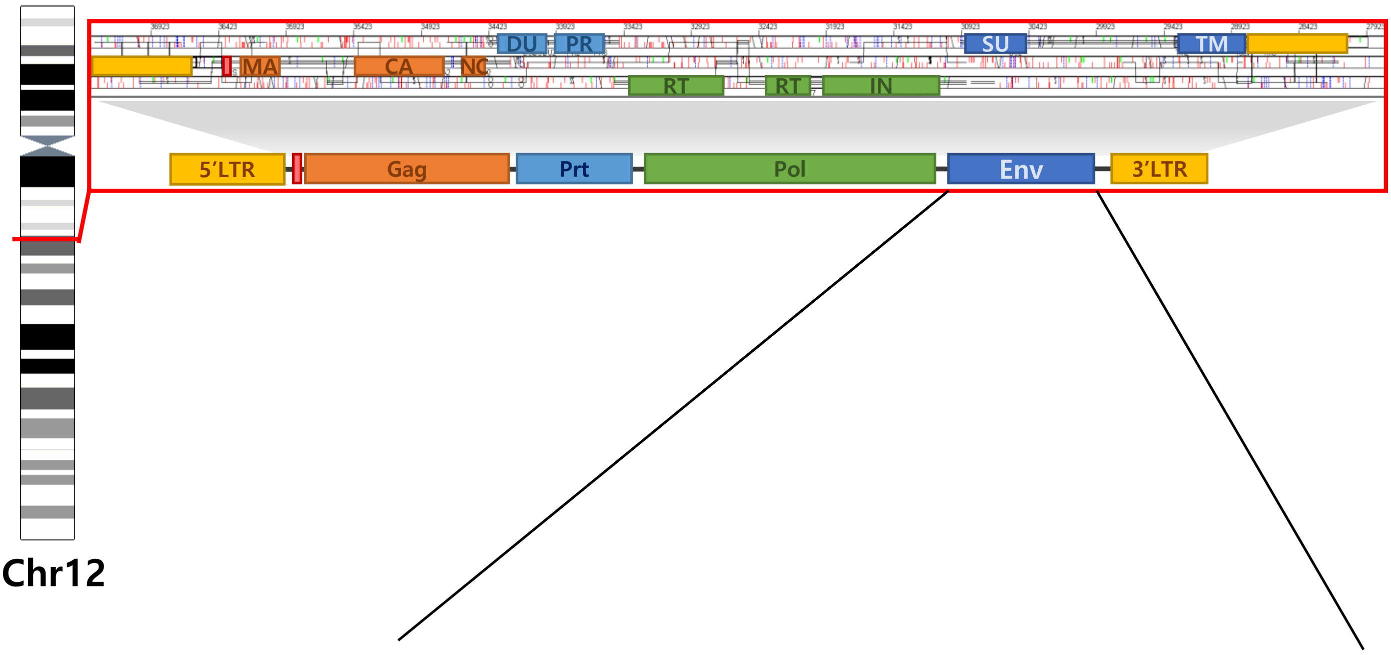

AGATTCATTTTTACTTTAATTGCAGTGATTATGGGATTAATTGCAGT
CACAGCTATGGCTGCTGTAGCAGGAGTTGCATTGCACTCTTTTGT
TCAGTCAGTAAACTTTGTTAATGATTGGCAAAAAAATTCTACAAGA
TTGTGGAATTCACAATCTAGTATTGATCAAAAATTGGCAAATCAAA

Guide RNA 1

TTAATGATCTTAGACAAA**CTGTCATTTGGATGGGAGACAGG**CTCA

Guide RNA 2

TGAGCTTAGAACATC**GTTTCCAGTTACAGTGTGACTGG**AATACGT

| | | | | | | | | | | | | | | | | | | | | | |

GGTCAATGTCACACTGACCTTAT

Guide RNA 3

CAGATTTTTGTATTACACCCCAAATTTATAATGAGTCTGAGCATCAC
TGGGACATGGTTAGACGCCATCTACAGGGAAGAGAAGATAATCTC
ACTTTAGACATTTCCAAATTAAAAGAACAAATTTTCGAAGCATCAAA
AGCCCATTTAAATTTGGTGCCAGGAACTGAGGCAATTGCAGGAGT
TGCTGATGGCCTCGCAAATCTTAACCCTGTCACTTGGGTTAAGAC
CATTGGAAGTACTACGATTATAAATCTCATATTAATCCTTGTGTGCC
TGTTTTGTCTGTTGTTAGTCTGCAGGTGTACCCAA

Supplement-Figure. 1 . Selection of three guide RNAs for targeting of HERV-K Env knockout system. Structure of HERV-K119 located on chromosome12 and the location of three guide RNAs for Crispr/Cas-9 kockout system of HERV-K *env* gene.

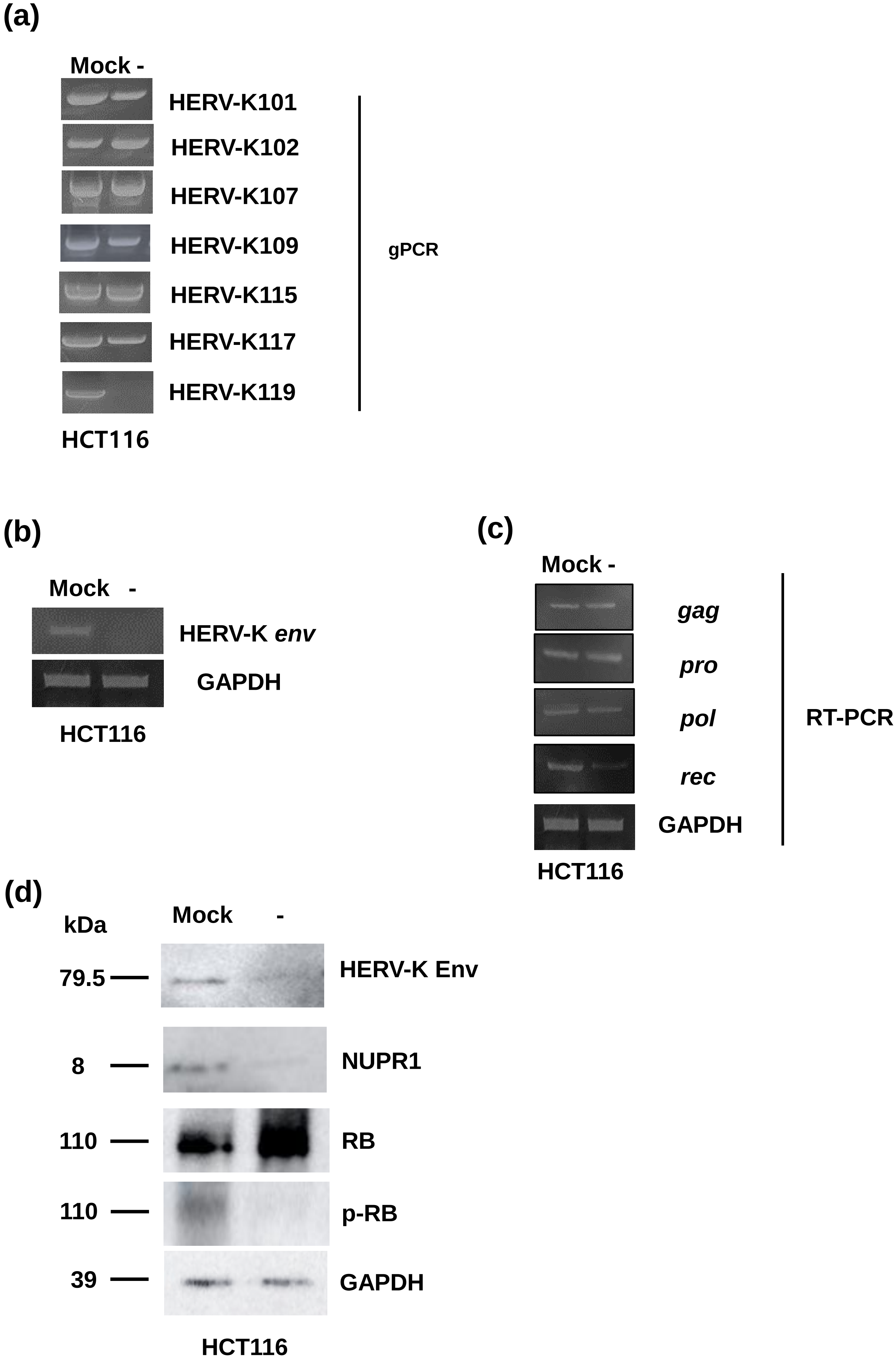

Supplement-Figure. 2 Knockout of HERV-K *env* gene in HCT116 colorectal cells. (a) Knockout of HERV-K *env* gene in HERV-K119 region. Genomic polymerase chain reaction (PCR) was performed for specific regions of HERV-K *env* derivatives. (b) Expression of HERV-K *env* RNA in HERV-K *env* knoutout (KO) in HCT116 cells. RT-PCR performed for general region of HERV-K *env* gene. (c) Expression of HERV-K119 *gag, pro, pol* and *rec* in HCT116 colorectal cancer cells. (d) Protein expression of various target genes regulated by HERV-K *env* gene. The expression of RB protein was significantly increased whereas phosphor-RB was decreased in HERV-K *env* KO HCT116 colorectal cancer cells.
